# Significant frequency of allelic imbalance in 3p region covering *RARβ* and *MLH1 loci* seems to be essential in molecular non-small cell lung cancer diagnosis

**DOI:** 10.1007/s12032-013-0532-9

**Published:** 2013-03-17

**Authors:** Adam Antczak, Monika Migdalska-Sęk, Dorota Pastuszak-Lewandoska, Karolina Czarnecka, Ewa Nawrot, Daria Domańska, Jacek Kordiak, Paweł Górski, Ewa Brzeziańska

**Affiliations:** 1 Department of General and Oncological Pulmonology, Medical University of Lodz, Kopcińskiego St.22, 90-153 Lodz, Poland; 2 1st Chair of Internal Diseases, Department of Molecular Bases of Medicine, Medical University of Lodz, Pomorska St. 251, 92-213 Lodz, Poland; 3 Department of Thoracic Surgery, General and Oncologic Surgery, Medical University of Lodz, Żeromskiego St. 113, 90-710 Lodz, Poland; 4 Department of Pneumology and Allergology, Medical University of Lodz, Kopcińskiego St. 22, 90-153 Lodz, Poland

**Keywords:** Non-small cell lung carcinoma (NSCLC), Loss of heterozygosity (LOH), Microsatellite instability (MSI), Microsatellite markers, Molecular diagnosis

## Abstract

The aim of the study was to investigate the influence of allelic imbalance (AI) in several *loci* of tumor suppressor genes in 3p region on the non-small cell lung cancer (NSCLC) development. We evaluated the frequency of loss of heterozygosity and/or microsatellite imbalance (LOH/MSI) and assessed their association with patients’ characteristics (age, gender, tobacco addiction) and NSCLC classification according to TNM/AJCC staging. To analyze the potential role of AI involved in NSCLC pathogenesis, we allelotyped a group of 74 NSCLC patients using 7 microsatellite markers. The highest frequency of LOH/MSI, however, not statistically significant, was observed in *RARβ* and *MLH1* (*p* = 0.104 and *p* = 0.216, respectively) *loci*. The association between high LOH/MSI frequency in 3p region with male gender (*p* = 0.041) as well as with age (especially >60 years) for *RARβ* and *MLH1* genes (*p* = 0.0001 and *p* = 0.020, respectively) was documented. Statistically significant increased frequency of *MLH1* allelic loss in squamous cell carcinoma (SCC) versus non-squamous cell carcinoma (non-SCC) was observed (*p* = 0.01). Significant increase in LOH/MSI frequency in 3p region (mainly in *FHIT* and *MLH1* *loci*) in correlation with cigarette addiction in a lifetime (≥40 years and ≥40 Pack Years) was also documented (*p* < 0.05). The highest LOH/MSI was revealed in *RARβ locus* in IA tumors (*p* = 0.0001), while the similarly high allelic loss of *MLH1* correlated with III A/B tumors (*p* = 0.0002), according to AJCC staging. The obtained results demonstrate that AI is influenced by tobacco smoking and seems to be vital in the molecular diagnosis of NSCLC, especially of SCC subtype.

## Introduction

Lung cancer is one of the leading cause of cancer mortality in most developed countries, especially in men between the ages of 50 and 70 [1, 2]. Based on the histological verification and tumor biology, lung cancer is classified into two major groups: (1) small cell lung carcinomas (SCLC), accounting for about 20 % and (2) non-small cell lung carcinoma (NSCLC), constituting approximately 75 % of all primary lung cancers. NSCLC is further divided into squamous cell carcinoma (SCC) and non-squamous cell carcinoma (non-SCC) with distinctive subtypes: adenocarcinoma (AC) and large cell carcinoma (LCC) [3]. This group of lung tumors is biologically heterogeneous, and the molecular studies of NSCLC, including genomic microarray and allelic imbalance analysis, are promising to extend and improve standard pathological methods of lung tumor assessment [4–6].

It has been documented that tobacco smoking is the most important risk factor in lung cancer development. Epidemiological studies in European populations have indicated that the increased incidence of lung cancer is proportional to the amount of smoked cigarettes and may have significant effect on tobacco-related cancer (TRC) risk [7–9]. However, it is well known that lung cancer (mainly NSCLC) is a genetically complex disease, developing in a result of the accumulation of multiple genetic abnormalities. Numerous genetic/epigenetic factors, especially gene polymorphisms, copy number alteration or gene methylation profiles, have been extensively investigated in lung cancer [7, 10–14]. The aggressive cancer phenotypes and their impact on patient outcome are actually characterized by numerous of these molecular changes [15–18]. Microsatellite instability (MSI) and more frequently occurring loss of heterozygosity (LOH) have been identified as the initial event in lung carcinogenesis and recognized in multiple chromosomal regions: 1p, 2p, 2q, 3p, 4q, 5q, 6p, 6q, 7q, 7p, 8p, 9p, 10q, 11p, 13p, 13q, 17p, 18q, 19q, 21q and 22q [16, 19–21]. Among them, the microsatellite alterations in 3p are regarded as biomarkers in genetic classification of pathological stages of NSCLC, as well as markers having prognostic significance [17, 18, 21]. However, despite many molecular studies on loss of heterozygosity and/or microsatellite imbalance (LOH/MSI) in 3p, the important tumor suppressor genes (TSGs) accepted as candidate genes associated with lung tumorigenesis have not been unequivocally identified yet. It is important to focus on this issue as inactivation of TSGs is a vital genetic event during the initiation as well as progression of lung carcinogenesis. The aim of our study was to confirm whether LOH/MSI alterations in the selected gene *loci* in 3p (*FHIT, RASSF1A,* *MLH1,* *RARβ,* *VHL*) might have important diagnostic and/or prognostic value in NSCLC patients.

## Materials and methods

### Biological material

The procedures used in the study were approved by the Ethical Committee of the Medical University of Lodz (RNN/140/10/KE). Informed written consent was received from each patient.

Lung tissue samples (100–150 mg) were obtained from patients (47 men and 27 women) who had undergone pulmonectomy or lobectomy at the Department of Thoracic Surgery, General and Oncologic Surgery, Medical University of Lodz, Poland, between July 2010 and June 2012. The studied biological material included 74 non-small cell lung carcinoma specimens and 74 matching macroscopically unchanged lung tissue samples received from the most distant site from the resected center of the primary lesion. Immediately after resection, tissue samples were collected in RNAlater^®^ and frozen at −80 °C. The resected NSCLC specimens were postoperatively histopathologically evaluated and classified according to the AJCC staging as well as TNM classification (pTNM), according to the WHO Histological Typing of Lung Tumor and IASCLC Staging Project 7th ed. (2010) Cancer [22].

### Characterization of the patients

The study involved 74 patients with diagnosed non-small cell lung carcinoma. The clinical characteristics of the studied patients, including their smoking habits, and histopathological verification of NSCLC samples are shown in Table 1. The smoking history was available for 71 patients: 5 patients were non-smokers, and 66 were smokers or former smokers. The amount of cigarettes smoked was presented as Pack Years (PYs) and was calculated according to the NCI Dictionary of Cancer Terms (1 Pack Year is equal to 20 cigarettes smoked per day for 1 year) [23].

**Table 1:** Clinical characteristics of the studied patients and histopathological verification of NSCLC

Clinical and pathological features	*n* (%)
Mean age (total), 65 ± 8.433 (range 47–87)	74
Men, 65 ± 8.129 (range 47–87)	47 (63.5)
Women, 63 ± 8.823 (range 42–79)	27 (36.5)
Age groups	
<60	20 (27)
60–70	32 (43)
>70	22 (30)
Smokers, *n* total = 71	66 (93)
Smoking period	
<40 years	37 (52)
≥40 years	29 (41)
Amount of cigarettes smoked	
10–15 cigarettes per day	6 (8)
20 cigarettes per day (1 pack)	43 (61)
30–40 cigarettes per day (1.5–2 packs)	17 (24)
Pack years (PYs)	
<40 PYs	30 (42)
≥40 PYs	36 (51)
Histopathological type of NSCLC	
Squamous cell carcinoma (SCC)	40 (54)
Non-squamous cell carcinoma (non-SCC)	34 (46)
Adenocarcinoma (AC)	27 (37)
Large cell carcinoma (LCC)	7 (9)
pTNM	
T1	20 (27)
T2	34 (46)
T3–4	20 (27)
AJCC	
AJCC IA	14 (19)
AJCC IB	14 (19)
AJCC IIA	14 (19)
AJCC IIB	9 (12)
AJCC IIIA/IIIB	23 (31)

### DNA extraction

Isolation of genomic DNA from NSCLC samples and matching macroscopically unchanged lung tissues (reference DNA) was performed using QIAamp DNA Mini Kit (Qiagen, Germany), according to the manufacturer’s protocol. The quality and quantity of isolated DNA was spectrophotometrically assessed (Eppendorf BioPhotometr™ Plus, Eppendorf, Germany). DNA with a 260/280 nm ratio in range 1.8–2.0 was considered to be of high quality and used in further analysis.

### Microsatellite analysis

The markers used for microsatellite analysis were selected from NCBI database (http://www.ncbi.nlm.nih.gov/genome/sts/sts) with supplementary mapping information, if necessary, provided in Cooperative Human Linkage Centre Database (http://www.chlc.org) or Genome Database (http://www.gdb.org). The chosen 7 microsatellite markers contained polymorphic microsatellite repeats, that is, (TG)n, (CA)n and (CAAA)n, and were linked to the chromosomal regions (3p14.2, 3p21.3, 3p22.2, 3p24.2 and 3p25.3) covering the *loci* of genes involved in significant processes of carcinogenesis, especially cell cycle regulation, proliferation and adhesion.

Pairs of DNA samples, that is, one sample obtained from the primary lesion and the other from unchanged lung tissue within the operational margin from the same patient, were amplified using primers for the studied microsatellite markers and AmpliTaq Gold^®^ 360 DNA Polymerase Kit (Applied Biosystems, USA). Reaction mixtures (total volume of 12.5 μl) contained 30–40 ng DNA, 10× AmpliTaq Gold^®^ 360 buffer (150 mM Tris–HCl, pH 8.3, 500 mM KCl), 360 GC Enhancer, 5 U/μl AmpliTaq Gold^®^ 360 DNA Polymerase, 25 mM MgCl_2_, 10 mM dNTPs, forward and reverse primers 0.5 μM each and nuclease-free water. All forward primers were labeled at 3′-end with fluorescent dye: 6-FAM, NED, PET or VIC. The temperatures of annealing were experimentally set for each pair of primers and were as follows: 45–47 °C (for D3S1317, D3S3611, D3S3615), 51–55 °C (for D3S1300, D3S1234) and 57–58 °C (for D3S1611, D3S1583). Negative and contamination controls were used for each marker. The chromosomal localization (region/gene) of the microsatellite markers and nucleotide sequences of primers used in the study are shown in Table 2.

**Table 2:** The chromosomal localization (region/gene) of the microsatellite markers, marker ID and nucleotide sequences of primers used in the study

Chromosomal region (gene)	Marker ID	Nucleotide sequence of primers (5′ → 3′)
3p14.2 (*FHIT*)	D3S1234	F^a^ CCTGTGAGACAAAGCAAGAC—F
		R^a^ GACATTAGGCACAGGGCTAA
	D3S1300	F AGCTCACATTCTAGTCAGCCT—F
		R GCCAATTCCCCAGATG
3p21.3 (*RASSF1A*)	D3S3615	F TGGAAAGGTAAGCACAAGC—N
		R TCCTCCCAGGAAGCAC
3p22.2 (*MLH1*)	D3S1611	F CCCCAAGGCTGCACTT—V
		R AGCTGAGACTACAGGCATTTG
3p24.2 (*RARβ*)	D3S1583	F AGCTTGTAAATAGGTCCTAACAGAG—N
		R TGGTTTAATAGGCACCGTTT
3p25.3 (*VHL*)	D3S1317	F TACAAGTTCAGTGGAGAACC—F
		R CCTCCAGGCCATACACAGTCA
	D3S3611	F GCTACCTCTGCTGAGCAT—V
		R TAGCAAGACTGTTGGGG

*F*, 6-FAM; *P*, PET; *N*, NED; *V*, VIC

^a^ *F* forward (sense), *R* reverse (antisense)

The quality of PCR products were analyzed in 2 % agarose gel electrophoresis, after bromide ethidium staining. In order to perform the capillary electrophoresis, 0.5 μl of PCR product was mixed with 0.25 μl GS500-LIZ Size Standard and Hi-Di™ Formamide (both reagents: Applied Biosystems, USA) up to the final volume of 10 μl. The obtained mixture was denatured for 5 min at 95 °C and subsequently cooled on ice for 3 min. The separation in capillary electrophoresis was conducted in 3130xl Genetic Analyzer (Applied Biosystems, Hitachi, USA), and the allele presence was assessed using GeneMapper Software v 4.0, according to the manufacturer’s protocol. The informativeness of the studied samples (heterozygosity) was confirmed when two distinct alleles were detected in the reference sample (DNA from unchanged lung tissue from the same patient). Evaluation of LOH/MSI was performed by calculating the ratio of the fluorescence intensity of the alleles from unchanged lung tissue sample (N, normal, that is, control sample) to the fluorescence intensity of the alleles from NSCLC sample (T, tumor). For each informative tumor–normal DNA pair (paired T and N samples), an allelic imbalance ratio (AIR) was calculated, based on the maximum allele peak heights (fluorescence intensity), as follows: normal allele 1:normal allele 2/tumor allele 1:tumor allele 2 (N1:N2/T1:T2), according to the previously published protocol [24]. LOH in tumor samples was considered indicative when AIR value was less than 0.67 or greater than 1.35 (according to the criteria of GeneMapper Software v 4.0). MSI in tumor DNA was considered indicative if one or more additional alleles were present in tumor DNA sample, as compared with the control DNA sample.

LOH/MSI frequency was calculated as a percentage of LOH/MSI alteration in relation to all informative *loci* (heterozygous DNA) and according to all analyzed tumors’ and patients’ variables.

### Statistical analysis

Chi-square test (*χ* ^2^) was used to assess the association between total LOH/MSI frequency (%) and chromosomal regions or markers. Nonparametrical statistical tests (Kruskal–Wallis or* U* Mann–Whitney test) were used to assess the association between total LOH/MSI frequency in 3p region and clinical variables of patients (age at diagnosis, gender, smoking habits), characteristics of the tumor (NSCLC histological subtype, tumor size according to TNM classification, tumor preoperative and postoperative staging according to AJCC classification). Statistical significance was determined at the level of *p* < 0.05. The results are presented as mean or median ± SEM and ±SD values. For calculations, Statistica for Windows v. 10 was applied.

## Results

### Allelic imbalance and microsatellite instability

Pairs of DNA specimens obtained from 74 NSCLC patients (cancerous and macroscopically unchanged tissue samples from each patient) were available for LOH/MSI analysis using a panel of 7 microsatellite markers. All studied DNA samples from NSCLC specimens were informative for at least two studied *loci*. The informativeness of the markers was assessed to be in the range of 42.66–90.66 % (mean 69.90 % ± 17.19). LOH/MSI changes were observed for all (7/7, 100 %) microsatellite markers.

**Fig. 1:**
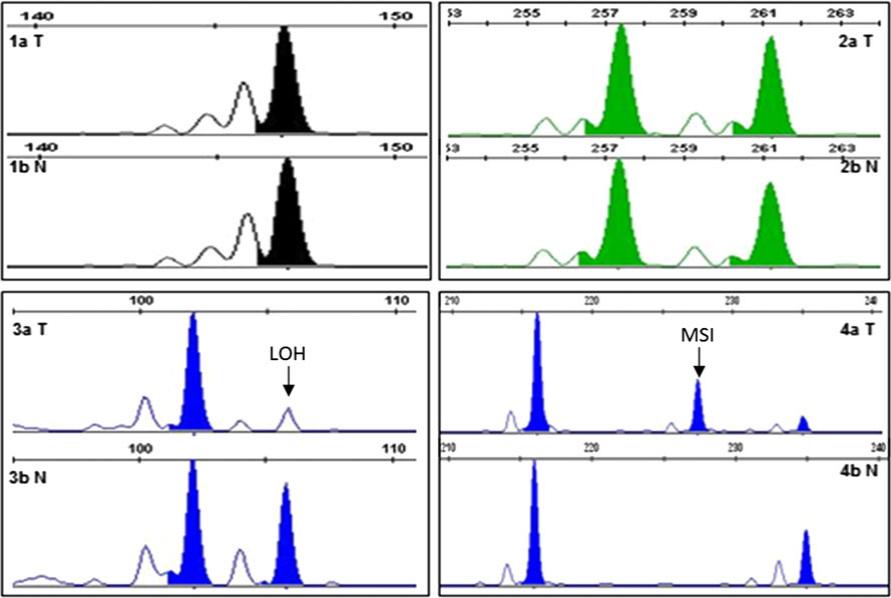
LOH/MSI analysis in NSCLC specimens (3130xl Genetic Analyzer, GeneMapper Software v. 4.0; Applied Biosystems, Hitachi).* 1a T*—homozygous DNA from tumor sample (sample no. 35, D3S1583 marker),* 1b N*—homozygous DNA from macroscopically unchanged lung tissue from the same patient,* 2a T*—heterozygous DNA from tumor sample (sample no. 12, D3S1611 marker),* 2b N*—heterozygous DNA from macroscopically unchanged lung tissue from the same patient,* 3a T*—LOH in DNA from tumor sample (sample no. 5, D3S1234 marker),* 3b N*—heterozygous DNA from macroscopically unchanged lung tissue from the same patient,* 4a T*—MSI in DNA from tumor sample (sample no. 72, D3S1300 marker),* 4b N*—heterozygous DNA from macroscopically unchanged lung tissue from the same patient

The obtained results indicate that in all studied samples, the frequency of LOH/MSI was in the range of 24.32–41.93 % (mean 33.85 % ± 6.17), depending on the marker. Representative examples of LOH/MSI in DNA derived from NSCLC samples are shown in Fig. 1.

### Total LOH/MSI frequency in particular *loci*

**Fig. 2:**
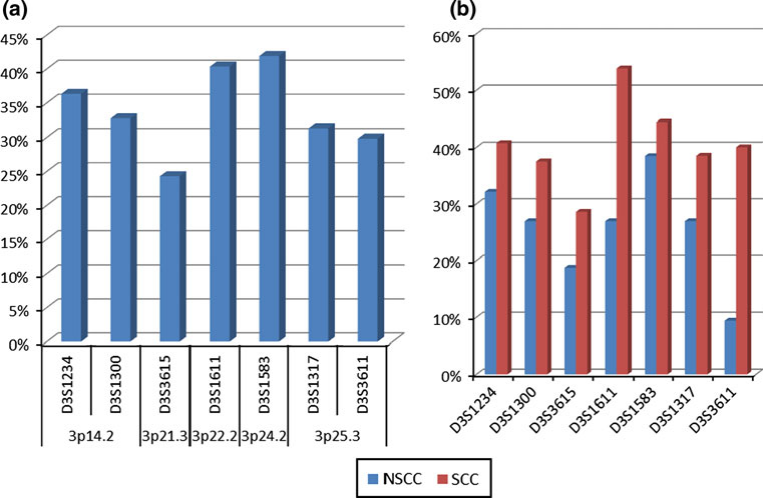
LOH/MSI frequencies (%) in NSCLC for all 7 studied microsatellite markers in **a** NSCLC samples; **b** in individual NSCLC histopathological subtypes (SCC vs. non-SCC)

Total LOH/MSI frequency (%) for each individual marker in NSCLC samples was assessed. The highest total frequency values were observed for D3S1583 marker (41.93 %; 13/31 informative *loci*) in 3p24.2 chromosomal region, that is, in *RARβ* *locus*, and for D3S1611 marker (40.38 %; 21/52 informative *loci*) in 3p22.2, that is, in *MLH1 locus*. The lowest LOH/MSI incidence (24.32 %; 9/37 informative *loci*) was identified for D3S3615 marker, localized in 3p21.3 (*RASSF1A locus*). Genetic instabilities of LOH/MSI type were also observed in *FHIT* *locus* for D3S1234 and D3S1300 markers, as well as in *VHL* *locus* for D3S1317 and D3S3611 markers, with similar frequencies: 36.36 % (20/55 informative *loci*), 32.76 % (19/58 informative *loci*), 31.37 % (19/60 informative *loci*) and 29.85 % (20/67 informative *loci*), respectively (see Fig. 2a).

Statistical analysis of LOH/MSI in particular gene *loci* revealed that the observed increase in LOH/MSI frequencies for D3S1583 (*RARβ*) and D3S1611 (*MLH1*) were not statistically significant (*p* = 0.104, χ^2^ = 2.65 and *p* = 0.216, χ^2^ = 1.53, respectively). The lowest frequency of total LOH/MSI observed for D3S3615 (*RASSF1A*) was also not statistically significant (*p* = 0.052; χ^2^ = 3.76).

### Correlation of total LOH/MSI frequency in 3p region with clinicopathological parameters

Total LOH/MSI frequency in 3p region was analyzed for all 7 markers in relation to histopathological characteristics of tumors (according to TNM and AJCC classifications and NSCLC subtypes) as well as clinical features of patients: gender, age at time of diagnosis.

Statistically significant differences in LOH/MSI frequency between all studied histopathological NSCLC subtypes (AC, LCC, SCC) were documented (*p* = 0.0006, ANOVA Kruskal–Wallis test). Taking into account a small number of LCC tumors (*n* = 7), we compared SSC versus non-SCC. The increased frequency of LOH/MSI in SCC group for all studied 3p *loci* was found (see Fig. 2b).

**Fig. 3:**
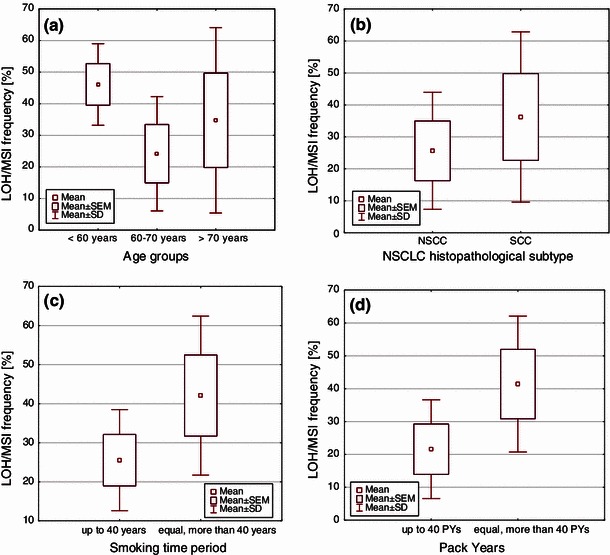
Box-and-whisker plots, representing mean LOH/MSI frequencies in the studied groups, according to: **a** age (*p* = 0.006, Kruskal–Wallis test); **b** NSCLC histopathological subtypes (*p* = 0.007,* U* Mann–Whitney test); **c** the period of smoking (*p* = 0.007,* U* Mann–Whitney test); **d** PYs (number of cigarettes smoked in a lifetime) (*p* = 0.004,* U* Mann–Whitney test)

Total LOH/MSI occurrence in 3p region was significantly more often in women as compared to men (*p* = 0.041;* U* Mann–Whitney test). Regarding patient’s age at time of diagnosis, they were divided into the following age groups: (1) up to 60 years, (2) 60–70 years, (3) over 70 years and significant differences were found between them (*p* = 0.006, ANOVA Kruskal–Wallis test). Statistically significant increase in LOH/MSI frequency was revealed in patients under the age of 60 years as compared with patients aged 60–70 years (*p* = 0.002,* U* Mann–Whitney test) (see Fig. 3a).

Statistical analysis confirmed the significantly higher LOH/MSI frequency in SCC group as compared with non-SCC (*p* = 0.007,* U* Mann–Whitney test) (see Fig. 3b).

No association was found between the total LOH/MSI frequency in 3p region and tumor clinical staging: pTNM classification (pT1, pT2, pT3–pT4), as well as AJCC classification (IA–B, IIA–B, IIIA–IIIB) (*p* > 0.05; Kruskal–Wallis test).

### Correlation of LOH/MSI frequency in particular *loci* with clinicopathological parameters

Loss of heterozygosity and/or microsatellite imbalance (LOH/MSI) frequency (%) was analyzed separately for each *locus* in relation to clinical features of patients: gender, patient’s age at time of diagnosis as well as histopathological characteristics of tumors (according to TNM and AJCC classifications and NSCLC subtypes).

The comparison of LOH/MSI frequencies between the studied microsatellites *loci* in men confirmed statistically significant low LOH/MSI frequency in 3p21.3 *locus* (D3S3615 marker; *RASSF1A*) (*p* = 0.0017, χ^2^ = 9.88) as compared with other markers.

Regarding the above mentioned age groups, statistically significant low LOH/MSI for D3S3615 marker (*RASSF1A*) in older patients, that is, at the age of 60–70 years (*p* = 0.0003, χ^2^ = 13.23) and >70 years (*p* = 0.0001, χ^2^ = 15.37), was found. In the same age groups, the highest LOH/MSI incidence was observed for D3S1611 (*MLH1*) and D3S1583 (*RARβ*) markers. Statistically significant high LOH/MSI frequency for D3S1611 marker (*MLH1*) in patients aged 60–70 years (*p* = 0.0198, χ^2^ = 5.43) and for D3S1583 marker (*RARβ*) in patients aged >70 years (*p* = 0.0001, χ^2^ = 29.98) were revealed.

According to TNM staging, the studied tumor samples were divided into three groups (pT1, pT2, pT3–T4). The highest frequency of LOH/MSI in the pT1 group was observed for D3S1234 marker (*FHIT*), and it was statistically significant (*p* = 0.0268, χ^2^ = 4.91).

Comparing the LOH/MSI frequencies in all specimens according to the AJCC classification (IA–B, IIA–B, IIIA–B), statistically significant high LOH/MSI frequencies for D3S1583 marker (*RARβ*) in AJCC IA–B group (*p* = 0.0001, χ^2^ = 24.04) and for D3S1611 marker (*MLH1*) in AJCC IIIA–B group (*p* = 0.0002, χ^2^ = 14.10) were found.

Statistically significant low LOH/MSI frequency was revealed for both D3S3615 (*RASSF1A; p* = 0.0018, χ^2^ = 9.73) and D3S3611 (*VHL*; *p* = 0.0001, χ^2^ = 18.35) markers in pT1 (TNM classification) and in IA–B group (AJCC classification; *RASSF1A*; *p* = 0.0015, χ^2^ = 10.06; *VHL* *p* = 0.034, χ^2^ = 4.50).

Regarding NSCLC histopathological subtypes (SCC and non-SCC), statistically significant high LOH/MSI frequency was observed for D3S1611 marker (*MLH1*) in SCC group (*p* = 0.011, χ^2^ = 6.47) and for D3S1583 marker (*RARβ*) in non-SCC group (*p* = 0.008, χ^2^ = 6.84).

### Total LOH/MSI frequency in 3p region and tobacco smoking

Regarding smoking history, patients were divided into two groups, taking into account the duration of smoking (<40 and ≥40 years), and the two other groups, taking into account the number of cigarettes smoked in a lifetime, assessed as PYs (<40 and ≥40 PYs). The increased total LOH/MSI frequency significantly correlated with the longest smoking history and with the increased PYs. Statistically significant increase in LOH/MSI frequency in 3p region was observed in case of patients who had been smoking for more than 40 years and more than 40 PYs (*p* = 0.007 and *p* = 0.004, respectively;* U* Mann–Whitney test) (see Fig. 3c, d).

### Total LOH/MSI frequency in particular *loci* and tobacco smoking

Total LOH/MSI frequency in each studied microsatellite *locus* considering patients’ smoking history, that is, the smoking period (<40 and ≥40 years) and the PYs (<40 and ≥40 PYs), was investigated.

Significantly high LOH/MSI frequency in 3p region, mainly for D3S1300 (*FHIT)* and D3S1611 (*MLH1)* markers in correlation with cigarette addiction in a lifetime ≥40 years (*p* = 0.015, χ^2^ = 5.88 and *p* = 0.009, χ^2^ = 6.82, respectively) and ≥40 PYs (*p* = 0.015, χ^2^ = 5.91 and *p* = 0.039, χ^2^ = 4.22, respectively), was found. Interestingly, statistically significant low LOH/MSI frequency for one marker, that is, D3S3615 (*RASSF1A*) was revealed in correlation with cigarette addiction in a lifetime ≥40 years (*p* = 0.020, χ^2^ = 5.35), as well as ≥40 PYs (*p* = 0.002, χ^2^ = 9.57). The results are shown in Table 3.

**Table 3:** LOH/MSI frequencies (%) in NSCLC patients regarding their smoking habits

Marker	D3S1234	D3S1300	D3S3615	D3S1611	D3S1583	D3S1317	D3S3611	*U* Mann–Whitney test
	LOH/MSI frequency (%)^a^							*p*
Smoking time period (years)								
<40	35	16	18	28	31	24	27	0.007
≥40	41	55	30	56	42	40	31	
Pack years (PYs)								
<40 PYs	33	8	19	21	21	26	22	0.004
≥40 PYs	41	54	25	52	45	37	34	

^a^Analyzed only in group of smokers

## Discussion

In our study, total LOH/MSI frequency in NSCLC samples was found in the range of 24–42 %, depending on the marker. The discrepancies between our results and those of others, who report 50–80 % frequency of LOH in NSCLC [25–27], may result from different markers used for LOH/MSI analysis, method of detection and evaluation of LOH/MSI, as well as from the population-based differences. However, we confirmed the implication of smoking as a very important causative factor in lung carcinogenesis supporting the hypothesis that cigarette smoking—both current and former—might induce molecular alterations in genes localized in 3p [27–30]. Additionally, we documented significantly higher total LOH/MSI frequency in SCC versus non-SCC subtype of lung cancer that is most closely associated to smoking.

Frequent allelic losses in 3p in lung carcinogenesis suggest the presence of multiple TSGs in this chromosomal region; however, only few genes have strong evidence supporting their candidacy as important in lung cancer [31, 32]. *RASS1A* gene, located in 3p21.3, frequently shows loss of expression in lung cancer cells. Two mechanisms of *RASSF1A* inactivation have been confirmed in lung tumors, namely LOH and promoter hypermethylation [26, 33]. Our study revealed rather low level of allelic loss (about 24 %) in *RASSF1A locus*, as compared to other studied genes. It could support the hypothesis of other investigators who suggest the lesser importance of LOH in *RASSF1A* inactivation in NSCLC tumorigenesis and the prevalence of epigenetic modifications [33, 34]. Our results may suggest the role of another, as yet unidentified, 3p21.3 TSG gene/s important in NSCLC. Indeed, as so far besides *RASSF1*, at least 7 other candidate TSGs (*CACNA2D2*, *PL6, 101F6, NPRL2*, *BLU, TUSC2* and *HYAL2*) have been identified in the 600-kb 3p21.3 homozygous deletion region [27, 32]. It still remains to be elucidated which gene/genes localized in this particular chromosomal region play a vital role and which mechanisms (promoter hypermethylation and/or LOH) resulting in TGS silencing are pivotal in lung carcinogenesis.

Another candidate gene in our study, *FHIT*, is located in the FRA3B fragile site at 3p14.2. Loss of *FHIT* expression is observed in lung cancer and pre-neoplastic lesions [30, 35, 36]. As found by Toledo et al. [37], LOH at *FHIT* gene in NSCLC is associated with high proliferation and low apoptotic level. In our study, LOH frequency in *FHIT* gene (33–36 %) was lower than that reported by others (44–58 %) [38, 39] however, we observed significant increase in LOH/MSI frequency in *FHIT* *locus* in correlation with cigarette addiction in a lifetime. It is in accordance with the observation of other authors and may support the hypothesis that cigarette smoking could induce molecular alterations of *FHIT* [29, 35]. Regarding other studied correlations, we did not recognize any associations between *FHIT* loss of heterozygosity and patient’s clinical features, outcome or metastatic behavior of tumor but we confirmed the association of frequent *FHIT* LOH with small size (pT1) of tumors, confirming the role of *FHIT* in the initiation of lung tumorigenesis. This is in agreement with the results of others [35, 40, 41].

Our analysis included also one of the genes implicated in the DNA mismatch repair system, that is, human MutL homolog (*hMLH1*), located in 3p22.2. Microsatellite instability in this chromosomal region is confirmed in lung cancer where it is recognized in 38–68 % of NSCLC patients [42, 43]. The results of our study confirm high frequency of *hMLH1* LOH (40.38 %). Based on our own findings and those of others, it may be concluded that AI in this *locus* (probably combined with epigenetic alteration) seems to be one of major events involved in lung carcinogenesis [43–45]. The significant correlation between *hMLH1* LOH and advanced stage of tumors (III A/B) found in our study might reflect the role of *hMLH1* in the late phase of carcinogenesis due to the accumulation of unrepaired DNA lesions. Additionally, our results indicate significantly increased frequency in AI in *hMLH1 locus* in patients (especially in SCC subtype) who smoke a lot. In fact, *hMLH1* reduced expression was more frequently associated with heavy smokers, assessed by daily tobacco uptake and total smoking exposure [42].

The gene encoding retinoic acid receptor beta (*RARβ*) has been found to be downregulated in lung tumors, suggesting its role lung carcinogenesis [46]. Frequent allelic losses in *RARβ* *locus* have been confirmed in lung tumors, NSCLC cell lines and in lung cancer precursor lesions [47, 48]. In our analysis, the frequency of LOH in *RARβ* *locus* was shown to be the highest among the studied loci, reaching nearly 42 %. This is in agreement with the results obtained by others [49]. Allelic loss in *RARβ* *locus* was also observed in smokers [47, 50]. In our study, we did not confirm this observation; however, the significant association between LOH in *RARβ locus* and stage I NSCLC found in our study might confirm the role of this gene in suppressing the lung tumorigenicity at its early stage.

The other candidate gene located in 3p region and included in our study is the von Hippel–Lindau (*VHL*) TSG. We used two markers flanking *VHL locus* (3p25.3). The observed frequency of AI found in the studied NSCLC samples, that is, about 30 %, appeared to be lower than that reported by others (46–63 %) [38, 39]. In some studies, higher LOH frequency was found in squamous cell carcinoma as compared with adenocarcinoma tumors [38, 51], although not confirmed in another study [39] neither in our analysis. Additionally, *VHL* LOH was more frequent in tumors from smokers as compared to those from non-smokers [38], which has not been confirmed in our study.

Reassuming, it should be stressed that respiratory epithelium carcinogenesis is a multifactorial process which includes inherited and acquired genetic changes (e.g., LOH/MSI), as well as epigenetic alterations. Additionally, cigarette smoking recognized as a one of the pivotal factors in lung cancer development, assessed in correlation with 3p region allelic losses provides controversial results. Therefore, the assessment of LOH/MSI in particular TSG *loci* separately and its correlation with smoking addiction might confirm the diagnostic and/or prognostic value of some genes (as in case of *RARβ* and *hMLH1* in our study) and the influence of cigarette smoking on gene alteration (*FHIT* and *hMLH1* in our study)—which seems to be promising.

## References

[1] Lung Cancer Incidence, mortality and prevalence Worldwide in 2008. http://globocan.iarc.fr/factsheet.asp.

[2] Greenlee RT, Murray T, Bolden S, Wingo PA (2000). Cancer statistics, 2000. CA Cancer J Clin.

[3] Ettinger DS, Akerley W, Bepler G (2010). Non-small cell lung cancer. J Natl Compr Cancer Netw..

[4] Garber ME, Troyanskaya OG, Schluens K (2001). Diversity of gene expression in adenocarcinoma of the lung. Proc Natl Acad Sci USA..

[5] Weir BA, Woo MS, Getz G (2007). Characterizing the cancer genome in lung adenocarcinoma. Nature.

[6] Broët P, Dalmasso C, Tan EH, et al. Genomic profiles specific to patient ethnicity in lung adenocarcinoma. Clin Cancer Res. 2011;17:3542–50. 10.1158/1078-0432.CCR-10-2185 21521776

[7] Mao L, Lee JS, Kurie JM (1997). Clonal genetic alterations in the lungs of current and former smokers. J Natl Cancer Inst.

[8] Lubin JH, Virtamo J, Weinstein SJ, Albanes D. Cigarette smoking and cancer: intensity patterns in the alpha-tocopherol, beta-carotene cancer prevention study in Finnish men. Am J Epidemiol. 2008;167:970–5. 10.1093/aje/kwm392 18250081

[9] Agudo A, Bonet C, Travier N, et al. Impact of Cigarette Smoking on Cancer Risk in the European Prospective Investigation into Cancer and Nutrition Study. J Clin Oncol. 2012;30:4550–7. 10.1200/JCO.2011.41.0183 23169508

[10] Souto-Garcia A, Fernández-Somoano A, Pascual T, Álvarez-Avellón SM, Tardón A (2012). Association of p21 Ser31Arg and p53Arg77Pro polymorphisms with lung cancer risk in CAPUA study. Lung Cancer Targets Ther..

[11] Staaf J, Isaksson S, Karlsson A, et al. Landscape of somatic allelic imbalances and copy number alterations in human lung carcinoma. Int J Cancer. 2012. doi: 10.1002/ijc.27879. 10.1002/ijc.27879 23023297

[12] Word B, Lyn-Cook LE Jr, Mwamba B, et al. Cigarette smoke condensate induces differential expression and promoter methylation profiles of critical genes involved in lung cancer in NL-20. Lung cells in vitro: short-term and chronic exposure. Int J Toxicol. 2012 [Epub ahead of print]. 10.1177/1091581812465902 23174910

[13] Yokota J, Shiraishi K, Kohno T. Genetic basis for susceptibility to lung cancer: Recent progress and future directions. Adv Cancer Res. 2010;109:51–72. 10.1016/B978-0-12-380890-5.00002-8 21070914

[14] Kettunen E, Salmenkivi K, Vuopala K (2006). Copy number gains on 5p15, 6p11-q11, 7p12, and 8q24 are rare in sputum cells of individuals at high risk of lung cancer. Lung Cancer..

[15] Chmara M, Wozniak A, Ochman K (2004). Loss of heterozygosity at chromosomes 3p and 17p in primary non-small cell lung cancer. Anticancer Res.

[16] Tseng RC, Chang JW, Hsien FJ (2005). Genomewide loss of heterozygosity and its clinical associations in non small cell lung cancer. Int J Cancer.

[17] Yoshino I, Fukuyama S, Kameyama T (2003). Detection of loss of heterozygosity by high-resolution fluorescent system in non-small cell lung cancer: association of loss of heterozygosity with smoking and tumor progression. Chest.

[18] Zhou X, Kemp BL, Khuri FR (2000). Prognostic implication of microsatellite alteration profiles in early stage non-small cell lung cancer. Clin Cancer Res.

[19] Balsara BR, Testa JR (2002). Chromosomal imbalances in human lung cancer. Oncogene.

[20] Girard L, Zochbauer-Muller S, Virmani AK (2000). Genomewide allelotyping of lung cancer identifies new regions of allelic loss, differences between small cell lung cancer and non-small cell lung cancer, and loci clustering. Cancer Res.

[21] Petersen S, Aninat-Meyer M, Schlüns K (2000). Chromosomal alterations in the clonal evolution to the metastatic stage of squamous cell carcinomas of the lung. Br J Cancer.

[22] Edge SB, Byrd DR, Compton CC, Edge SB, Byrd DR, Compton CC, Fritz AG, Greene FL, Trotti A (2010). Lung. AJCC cancer staging manual.

[23] http://www.cancer.gov/dictionary?CdrID=306510.

[24] Czarnecka K, Pastuszak-Lewandoska D, Migdalska-Sek M (2011). Aberrant methylation as a main mechanism of TSGs silencing in PTC. Front Biosci (Elite Ed)..

[25] Schayek H, Krupsky M, Yaron P (2006). Genetic analyses of non-small cell lung cancer in Jewish Israeli patients. Isr Med Assoc J..

[26] Dammann R, Li C, Yoon JH (2000). Epigenetic inactivation of a RAS association domain family protein from the lung tumour suppressor locus 3p21.3. Nat Genet.

[27] Wistuba II, Behrens C, Virmani AK (2000). High resolution chromosome 3p allelotyping of human lung cancer and preneoplastic/preinvasive bronchial epithelium reveals multiple, discontinuous sites of 3p allele loss and three regions of frequent breakpoints. Cancer Res.

[28] Castagnaro A, Marangio E, Verduri A (2007). Microsatellite analysis of induced sputum DNA in patients with lung cancer in heavy smokers and in healthy subjects. Exp Lung Res.

[29] Zienolddiny S, Ryberg D, Arab MO (2001). Loss of heterozygosity is related to p53 mutations and smoking in lung cancer. Br J Cancer.

[30] Sozzi G, Sard L, De Gregorio L (1997). Association between cigarette smoking and FHIT gene alterations in lung cancer. Cancer Res.

[31] Hibi K, Takahashi T, Yamakawa K (1992). Three distinct regions involved in 3p deletion in human lung cancer. Oncogene.

[32] Lerman MI, Minna JD (2000). The 630-kb lung cancer homozygous deletion region on human chromosome 3p21.3: identification and evaluation of the resident candidate tumor suppressor genes. The International Lung Cancer Chromosome 3p21.3 Tumor Suppressor Gene Consortium. Cancer Res.

[33] Agathanggelou A, Honorio S, Macartney DP (2001). Methylation associated inactivation of RASSF1A from region 3p21.3 in lung, breast and ovarian tumours. Oncogene.

[34] Seng TJ, Currey N, Cooper WA, et al. DLEC1 and MLH1 promoter methylation are associated with poor prognosis in non-small cell lung carcinoma. Br J Cancer. 2008;99:375–82. 10.1038/sj.bjc.6604452 PMC2480971 18594535

[35] Tseng JE, Kemp BL, Khuri FR (1999). Loss of FHIT is frequent in stage I non-small cell lung cancer and in the lungs of chronic smokers. Cancer Res.

[36] Tomizawa Y, Nakajima T, Kohno T (1998). Clinicopathological significance of FHIT protein expression in stage I non-small cell lung carcinoma. Cancer Res.

[37] Toledo G, Sola JJ, Lozano MD (2004). Loss of FHIT protein expression is related to high proliferation, low apoptosis and worse prognosis in non-small-cell lung cancer. Mod Pathol.

[38] Ho WL, Chang JW, Tseng RC (2002). Loss of heterozygosity at loci of candidate tumor suppressor genes in microdissected primary non-small cell lung cancer. Cancer Detect Prev.

[39] An Q, Liu Y, Gao Y (2002). Deletion of tumor suppressor genes in Chinese non-small cell lung cancer. Cancer Lett.

[40] Lee YC, Wu CT, Shih JY (2004). Frequent allelic deletion at the FHIT locus associated with p53 overexpression in squamous cell carcinoma subtype of Taiwanese non-small-cell lung cancers. Br J Cancer.

[41] Geradts J, Fong KM, Zimmerman PV, Minna JD (2000). Loss of FHIT expression in non-small-cell lung cancer: correlation with molecular genetic abnormalities and clinicopathological features. Br J Cancer.

[42] Xinarianos G, Liloglou T, Prime W (2000). hMLH1 and hMSH2 expression correlates with allelic imbalance on chromosome 3p in non-small cell lung carcinomas. Cancer Res.

[43] Wang YC, Lu YP, Tseng RC (2003). Inactivation of hMLH1 and hMSH2 by promoter methylation in primary non-small cell lung tumors and matched sputum samples. J Clin Invest..

[44] Chang JW, Chen YC, Chen CY (2000). Correlation of genetic instability with mismatch repair protein expression and p53 mutations in non-small cell lung cancer. Clin Cancer Res.

[45] Geng X, Wang F, Zhang L, Zhang WM (2009). Loss of heterozygosity combined with promoter hypermethylation, the main mechanism of human MutL Homolog (hMLH1) gene inactivation in non-small cell lung cancer in a Chinese population. Tumor..

[46] Brabender J, Metzger R, Salonga D (2005). Comprehensive expression analysis of retinoic acid receptors and retinoid X receptors in non-small cell lung cancer: implications for tumor development and prognosis. Carcinogenesis.

[47] Martinet N, Alla F, Farré G (2000). Retinoic acid receptor and retinoid X receptor alterations in lung cancer precursor lesions. Cancer Res.

[48] Virmani AK, Rathi A, Zöchbauer-Müller S (2000). Promoter methylation and silencing of the retinoic acid receptor-beta gene in lung carcinomas. J Natl Cancer Inst.

[49] Picard E, Seguin C, Monhoven N (1999). Expression of retinoid receptor genes and proteins in non-small-cell lung cancer. J Natl Cancer Inst.

[50] Ayoub J, Jean-François R, Cormier Y (1999). Placebo-controlled trial of 13-cis-retinoic acid activity on retinoic acid receptor-beta expression in a population at high risk: implications for chemoprevention of lung cancer. J Clin Oncol.

[51] Miyakis S, Liloglou T, Kearney S (2003). Absence of mutations in the VHL gene but frequent loss of heterozygosity at 3p25-26 in non-small cell lung carcinomas. Lung Cancer..

